# A five-year retrospective study on the epidemiology of hand, foot and mouth disease in Sabah, Malaysia

**DOI:** 10.1038/s41598-021-96083-3

**Published:** 2021-09-08

**Authors:** Siat Yee Fong, Daisuke Mori, Christina Rundi, Jun Fai Yap, Muhammad Jikal, A. L. Liza Binti Abd Latip, Victor Johnny, Kamruddin Ahmed

**Affiliations:** 1 Borneo Medical and Health Research Centre, Faculty of Medicine and Health Sciences, Universiti Malaysia Sabah, Jalan UMS, 88400 Kota Kinabalu, Sabah Malaysia; 2 Department of Biomedical Sciences, Faculty of Medicine and Health Sciences, Universiti Malaysia Sabah, Jalan UMS, 88400 Kota Kinabalu, Sabah Malaysia; 3 Department of Pathology and Microbiology, Faculty of Medicine and Health Sciences, Universiti Malaysia Sabah, Jalan UMS, 88400 Kota Kinabalu, Sabah Malaysia; 4 Sabah State Health Department, Ministry of Health Malaysia, Jalan Mat Salleh, 88590 Kota Kinabalu, Sabah Malaysia; 5 Beaufort Health Office, Ministry of Health Malaysia, Pekan Beaufort, 89800 Beaufort, Sabah Malaysia

**Keywords:** Infectious diseases, Epidemiology, Public health

## Abstract

Hand, foot, and mouth disease (HFMD) is endemic in Malaysia, with the number of cases increasing. Sabah has experienced several HFMD outbreaks, but information on the epidemiology and molecular characteristics of responsible viruses is scarce. In this study, data of 17,574 reports of HFMD cases in Sabah from 2015 to 2019 were extracted from a public health disease surveillance system and analyzed. Twenty-one swab samples from 13 children were collected from Beaufort, Sabah, during an outbreak in August 2018 for detection and serotyping of causative viruses by semi-nested reverse transcription-polymerase chain reaction (snRT-PCR) of the VP4–VP2 region and consensus degenerate hybrid oligonucleotide primer PCR of the VP1 region, respectively. Nucleotide sequencing and phylogenetic analysis were conducted by the neighbor-joining method. The average annual incidence of HFMD was 94.3 per 100,000 people, with the greatest yearly increase between 2017 and 2018. Swabs from six children were tested positive for enterovirus, of which five were positive for CVA16 and one for EV71. All CVA16 strains belonged to sub-genotype B1a, and the EV71 strain belonged to sub-genotype B5. Phylogenetic analyses indicate that enterovirus genotype shift might be responsible for the increasing trend of HFMD in Sabah, however, further study is needed.

## Introduction

Hand, foot, and mouth disease (HFMD) is a contagious viral infection. It commonly affects children under five years of age, but it can occasionally affect adults^1^. Symptoms of HFMD include fever, sore throat, and maculopapular or vesicular rashes on hands, feet, and mouth. Although the disease is generally mild, severe HFMD can lead to fatal neurologic and systemic complications^2^. HFMD is caused by a few members of the genus *Enterovirus*, belonging to the family *Picornaviridae*, particularly coxsackievirus A16 (CVA16) and enterovirus 71 (EV71) of the enterovirus A species^3^.

EV71 is divided into seven genotypes (A-G) based on the nucleotide diversity of the viral protein 1 (VP1) gene. Genotype A contains the prototype strain BrCr, which was first isolated in the United States in 1969. Genotypes B and C are more widely circulated throughout the world, while genotypes E–F and D–G were identified more recently in Africa and India, respectively^4^. CVA16 was first isolated in South Africa in 1951^5^. Due to the diversity of the VP1 gene, it is classified into two genotypes, A and B, where the latter is the predominant circulating genotype responsible for CVA16 infection worldwide^5^. Genotype B can be divided into B1 and B2, and genotype B1 can be further divided into B1a, B1b, and B1c^6^.

Major EV71-associated HFMD outbreaks have been occurring across the Asia–Pacific region since the late 1990s^7^. In Malaysia, the first outbreak due to EV71 infection was documented in Sarawak (East Malaysia) in April 1997 and subsequently spread to Peninsular Malaysia in June 1997, where more than 4000 children were affected and 41 deaths were reported^8,9^. CVA16 was also isolated from the HFMD outbreak in 1997, but only EV71 was isolated from fatal cases^9^. CVA16 infection was seen to occur during the outbreak or inter-outbreak of EV71^10^.

Several researchers have characterized EV71 and CVA16 strains from HFMD cases in Peninsular Malaysia and Sarawak^10–13^. Although a number of HFMD outbreaks have been reported in Sabah, information on the genetic characteristics of these strains in Sabah is limited. So far, only two studies have been conducted on EV71 strains isolated from Sabah in 1999^11^ and 2008^12^, but none on CVA16. Since then, no studies have been conducted on HFMD in Sabah for almost a decade. The limited research could be due to the assumption of low burden of HFMD in Sabah and a lack of standardized sample collection protocols that would maximize the yield of viruses for molecular characterization. More surveillance is necessary to understand and monitor the emergence and spread of new strains. Besides, understanding the epidemiological pattern of HFMD is important for the implementation of appropriate intervention strategies. Therefore, this five-year retrospective study explored the epidemiological pattern of HFMD in Sabah using the data of 17,574 reports of HFMD cases recorded in a Malaysian public health disease surveillance system, e*Notifikasi*, and investigated the genotype characteristics of seven enterovirus-positive samples that caused HFMD in the region.

## Results

### Temporal and demographic trends

**Figure 1:**
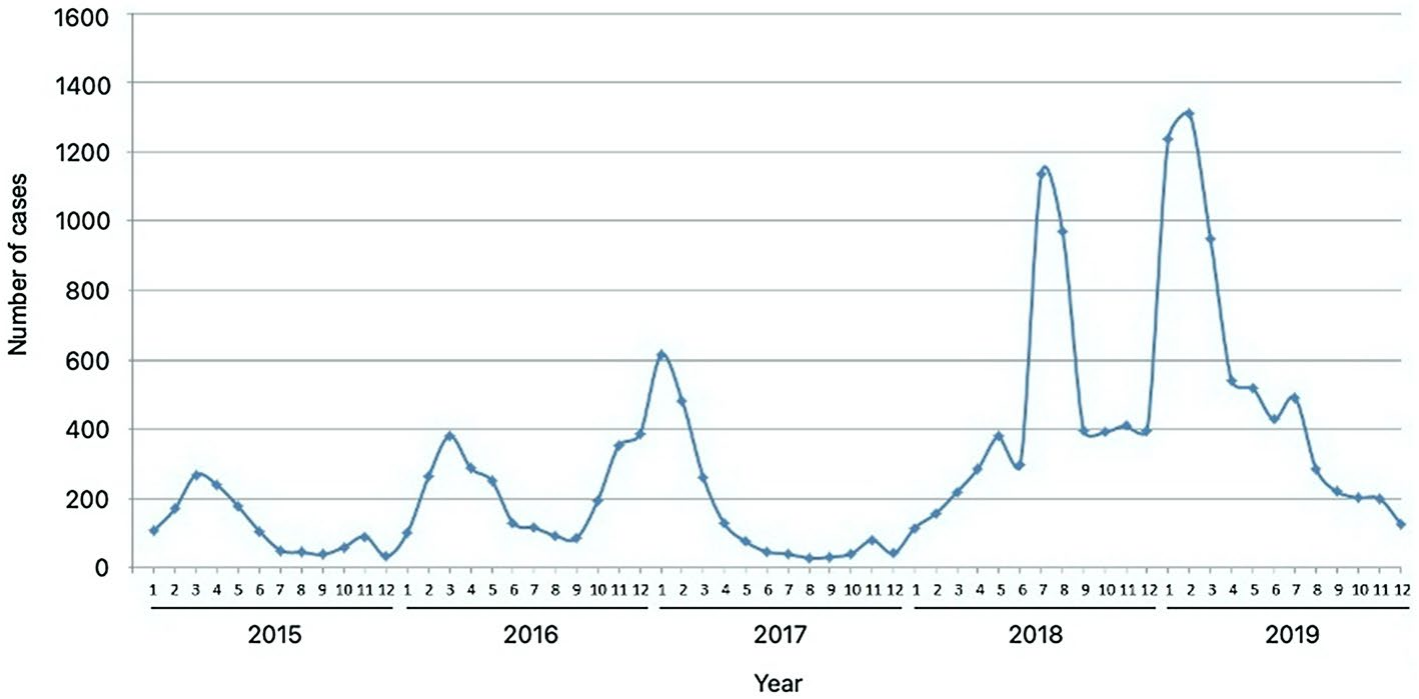
Monthly distribution of hand, foot, and mouth disease cases in Sabah from 2015 to 2019.

From the *eNotifikasi* system (http://enotifikasi.moh.gov.my), a total of 17,574 reports of HFMD cases were recorded in Sabah during the five-year study period, with no deaths reported. While cases were notified year-round, seasonal analysis showed that reported cases peaked annually between January and March, except in 2018, where HFMD peaked in July (Fig. 1). The number of cases gradually increased from 2015 to 2019, but there was a slight decrease in 2017 from the previous year (Table 1). The annual incidence rate from 2015 to 2019 varied from 39.9 per 100,000 people to 166.1 per 100,000 people, with a five-year average annual incidence of 94.3 per 100,000 people. The greatest year-to-year increase, approximately 2.7-fold, was observed from 2017 to 2018. Notably, similar trends for gender and age groups were observed every year. Males accounted for most of the cases (male-to-female ratio of 1.3:1), and one-year-old children had the highest number of cases for both genders.

**Table 1:** Demographic characteristics of hand, foot, and mouth disease cases in Sabah, Malaysia, from 2015 to 2019.

Year	2015	2016	2017	2018	2019 Total
No. of cases	1390	2644	1872	5157	6511
Incidence rate (per 100,000)	39.9	75.9	51.1	138.6	166.1
**Gender**					
Male	769 (55.3)	1464 (55.4)	1054 (56.3)	2930 (56.8)	3671 (56.4)
Female	621 (44.7)	1180 (44.6)	818 (43.7)	2227 (43.2)	2840 (43.6)
**Age in years**					
< 1	155 (11.2)	217 (8.2)	183 (9.8)	468 (9.1)	601 (9.2)
1	365 (26.3)	673 (25.5)	575 (30.7)	1254 (24.3)	1743 (26.8)
2	282 (20.3)	515 (19.5)	362 (19.3)	969 (18.8)	1313 (20.2)
3	180 (12.9)	423 (16.0)	261 (13.9)	697 (13.5)	906 (13.9)
4	136 (9.8)	278 (10.5)	178 (9.5)	533 (10.3)	710 (10.9)
5	101 (7.3)	183 (6.9)	109 (5.8)	414 (8.0)	490 (7.5)
> 5	171 (12.3)	355 (13.4)	204 (10.9)	822 (15.9)	748 (11.5)
**Transmission classification**					
Clusters of cases	121 (8.7)	143 (5.4)	88 (4.7)	990 (19.2)	696 (10.7)
Sporadic cases	1269 (91.3)	2501 (94.6)	1784 (95.3)	4167 (80.8)	5815 (89.3)

Percentage of total is shown in parenthesis.

### Spatial trends across districts

**Figure 2:**
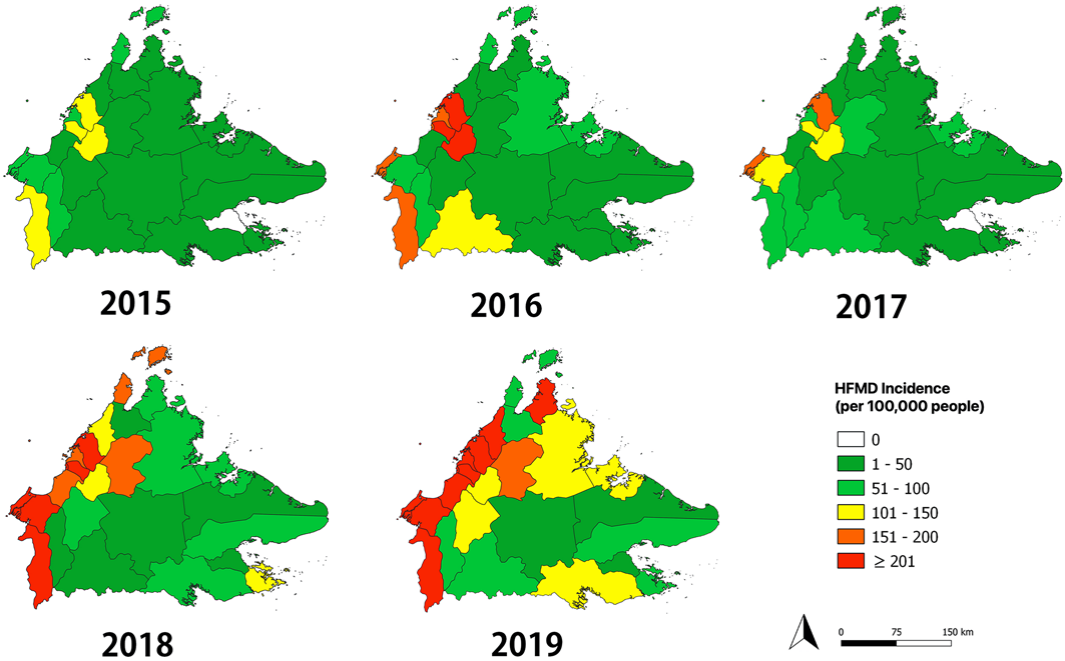
Annual spatial trend of hand, foot, and mouth disease across the 25 districts in Sabah from 2015 to 2019. The maps were generated using QGIS version 3.14.1 Pi (https://www.qgis.org/).

Among the 25 districts, Kota Kinabalu reported the highest number of HFMD cases every year (total 3986 cases), with a peak of 1328 cases in 2019 (Table 2). On the contrary, Tongod district had the lowest number of cases, with just 19 cases reported throughout the five years. Of the 25 districts, a peak in the number of cases was observed in 17 (68%) districts in 2019, five (20%) districts in 2018, two (8%) districts in 2016, and one (4%) district in 2015. The annual spatial trend dynamics are shown in Fig. 2, indicating that there was a yearly variation across districts. Throughout the five-year study period, the district with the largest variation in annual incidence rate was Beaufort (58 per 100,000 people in 2016 to 657 per 100,000 people in 2018) while Kinabatangan had the smallest variation in annual incidence rate, ranging from 8 per 100,000 people in 2015 to 18 per 100,000 in 2018. A prominent spatial change was observed in 2018, when incidence increased markedly in the western regions of Sabah from the previous year. High incidence rates were not only found in densely populated districts, such as Kota Kinabalu, Putatan and Penampang, but also in low population density districts, including Sipitang, Beaufort, Kuala Penyu and Kota Belud. The highest incidence district was Kuala Penyu with a five-year average annual incidence of 257 per 100,000 people, while the lowest were Tongod and Kunak districts, both with 9 per 100,000 people.

**Table 2:** The number of hand, foot, and mouth disease cases in each district of Sabah from 2015 to 2019.

Districts	Year					Total
	2015	2016	2017	2018	2019	
Kota Kinabalu	427	771	431	1029	1328	3986
Papar	12	9	3	326	642	992
Tuaran	152	288	200	344	380	1364
Ranau	7	26	65	185	224	507
Kota Belud	3	8	13	150	242	416
Penampang	201	352	189	484	582	1808
Putatan	44	111	46	146	198	545
Kudat	58	64	29	142	77	370
Kota Marudu	4	3	11	37	71	126
Pitas	2	16	5	29	109	161
Sandakan	33	217	236	365	535	1386
Beluran	48	92	44	97	158	439
Tongod	6	3	5	1	4	19
Kinabatangan	13	20	16	31	27	107
Tawau	125	166	84	435	697	1507
Semporna	4	2	5	160	88	259
Kunak	0	3	0	15	17	35
Lahad Datu	14	66	108	150	212	550
Beaufort	66	49	88	515	219	937
Sipitang	50	70	31	147	204	502
Kuala Penyu	20	36	39	118	105	318
Keningau	4	74	97	168	253	596
Tenom	49	49	53	26	56	233
Tambunan	43	112	46	45	60	306
Nabawan	5	37	28	12	23	105

### Phylogenetic analysis of enteroviruses

A total of 21 swab samples were collected from 13 children during an outbreak reported in Beaufort, Sabah, in August 2018. Swabs from six children comprising of four females and two males, ranging from one to seven years old were tested positive for enteroviruses using snRT-PCR.

Sequence analysis of the VP1 gene of the positive snRT-PCR samples by BLAST identified five (83.3%) CVA16 and one (16.7%) EV71 serotypes. The partial VP1 sequences of the CVA16 and EV71 viruses were 254 bp and 359 bp in length, respectively. These partial VP1 sequences were used to construct the phylogenetic trees of the two serotypes.

**Figure 3:**
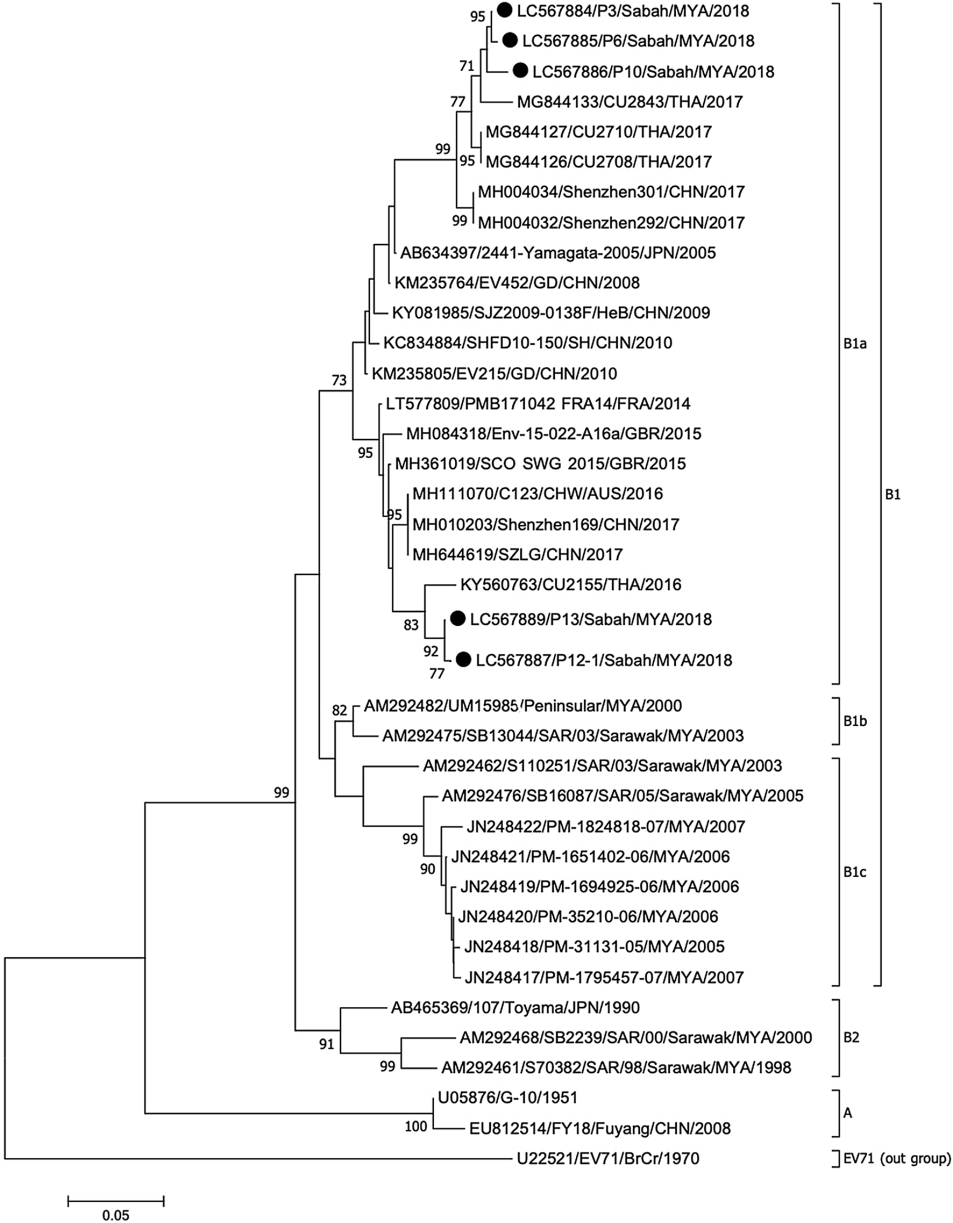
Phylogenetic tree constructed using partial VP1 sequences (254 bp) of coxsackievirus A16 strains by neighbor-joining method. The strains analyzed in this study are marked with a filled circle. The number adjacent to the node represents the bootstrap value; values < 70% are not shown. The scale bar at the bottom indicates the genetic distance expressed as nucleotide substitutions per site. The nucleotide sequences of our strains have been submitted to the DNA Data Bank of Japan (DDBJ) with accession nos. LC567884–LC567887 and LC567889.

This is the first study that shows the genetic characteristics of CVA16 strains from Sabah. According to the phylogenetic analysis, all five CVA16 strains detected in this study were classified as genotype B, specifically sub-genotype B1a. Three CVA16 strains clustered with Thai strains from 2017, while the other three clustered with another Thai strain from 2016 (Fig. 3). None of the CVA16 strains in this study belong to the same sub-genotypes as the other Malaysian strains, namely B1b, B1c and B2. The circulating strains in Malaysia in the early 2000s were of sub-genotypes B1b and B2, which were later replaced with B1c as the predominant sub-genotype in the late 2000s. However, more studies are needed to determine the evolutionary change of CVA16 in Malaysia.

**Figure 4:**
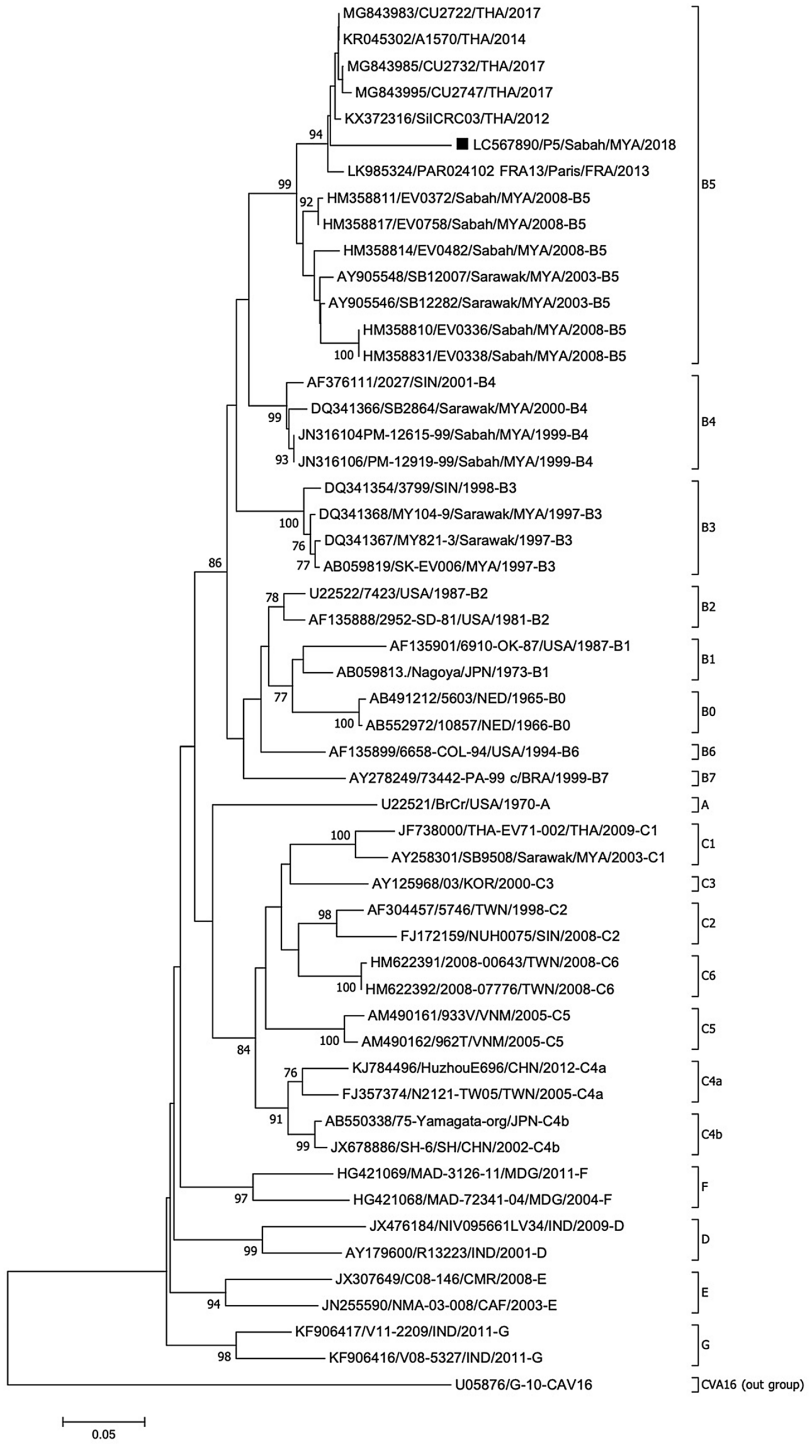
Phylogenetic tree constructed using partial VP1 sequences (359 bp) of enterovirus 71 strains by neighbor-joining method. The strain analyzed in this study is marked with a filled square. The number adjacent to the node represents the bootstrap value; values < 70% are not shown. The scale bar at the bottom indicates the genetic distance expressed as nucleotide substitutions per site. The nucleotide sequence of our strain has been submitted to the DNA Data Bank of Japan (DDBJ) with accession no. LC567890.

The only EV71 strain detected in this study was classified as a genotype B, precisely sub-genotype B5 (Fig. 4). The strain was clustered closely together with five EV71 strains from Thailand detected in 2012, 2014, and 2017, and a strain from France detected in 2013. However, it did not cluster with any previously detected Malaysian strains. Strains isolated from Sarawak and Sabah in 2003 and 2008, respectively, also belonged to the same sub-genotype but of different lineage. Sub-genotype B4 was found circulating in Sabah and Sarawak in 1999 and 2000, respectively, while B3 was the predominant sub-genotype during the 1997 outbreak in Sarawak. From this phylogenetic analysis, genotypic changes of EV71 have been observed over time even in the same region, for instance Sarawak.

## Discussion

There has been a considerable increase in reports of HFMD cases in Sabah throughout the five-year study period. HFMD cases peaked in the early months, between January and March every year, except in 2018, where the highest number of reported cases was in July. The exact causes of the increment of cases and the notable seasonal characteristic of HFMD as well as the different peak period in 2018 are unclear. However, there are several factors that could possibly affect HFMD incidence.

A study by Zhao and Hu (2019) found that school terms and a major public holiday in China, the Chinese Spring Festival, affected the HFMD transmission seasonality in mainland China, contributing to two major waves in the region^14^. The study revealed that the Chinese Spring Festival reflected the seasonal contact rate in the population, which dominantly caused the larger peak in March, while the school terms reflected the seasonal contact rate in children, resulting in the smaller peak in autumn^14^. The school year in Malaysia starts in early January and it is divided into two school terms: from January to May for the first term and from June to November for the second term. Besides, Chinese New Year, which is the same as the Chinese Spring Festival, is one of Malaysia’s biggest holidays celebrated in the beginning of the year, between late January and mid-February. The first day of school and the Chinese New Year celebration in Malaysia when combined, could be a driving factor for the seasonal peaks (between January and March every year, except for 2018) revealed in this study. However, further in-depth research is necessary to determine the effects of school terms and holidays on HFMD transmission in Malaysia.

In 2018, a large HFMD outbreak was reported in Malaysia, which affected over 76,000 children nationwide^15^. Selangor recorded the highest number of HFMD cases on August 4, 2018 with 12,868 cases, followed by Kuala Lumpur with 4996 cases and Sarawak with 4988 cases^16^. Although the number of HFMD cases in Sabah was not as high as these states, the outbreak undoubtedly had a significant impact on the local community as the region in general, experienced a sudden spike in HFMD cases in 2018. As shown in this study, cases in 2018 continued to soar upwards until July of the same year, unlike the previous years and 2019, where cases started to decrease after March, which could be due to exhaustion of susceptible hosts^14^ or effect of control measures. A possible cause for the unusual pattern in 2018 might be due to the movement of people traveling to their respective locations for polling during Malaysia’s 14th General Election on May 9, 2018. This was later followed by two major celebrations, the Harvest Festival, which is an important regional event and Eid-ul-Fitr (Festival of Breaking the Fast) celebrated in late May and mid-June, respectively.

Meteorological factors, such as air temperature, rainfall, relative humidity, air pressure and wind speed, have been shown to impact HFMD differently depending on the climatic zones and latitudes^17,18^. A meta-analysis by Duan et al. (2019), revealed that HFMD incidences in subtropical and temperate regions were significantly associated with meteorological factors but not in tropical regions^17^. On the contrary, other studies of tropical climate found significant correlation between HFMD and weather variables, including air temperature, rainfall, pressure, and wind speed^19,20^. These show that meteorological factors have inconsistent and varying effects even in regions with the same climate, However, the effects of meteorological factors on HFMD incidence in this study were not examined and thus, the question on whether these factors contribute to the seasonal peaks in Sabah remains an enigma.

In this study, CVA16 and EV71 were detected from the swab samples collected during the HFMD outbreak in Beaufort in August 2018, where the former was the predominant serotype. Other recent studies of HFMD in Peninsular Malaysia also reported the presence of CVA16 and EV71 in their samples, besides CVA6, which was not detected in this study. Ling et al. (2014) identified CVA6, CVA16 and EV71 from specimens collected from patients in Seri Kembangan, Selangor, where CVA6 was the most common among the three types of enterovirus^10^. Another study by Lee et al. (2021) reported that only CVA6 and CVA16 were found in clinically diagnosed HFMD patients in Kuala Lumpur and Selangor, where CVA6-infected cases were more common in children under 12 months old^15^.

EV71 and CVA16 are regarded as the two major etiological agents of HFMD in Malaysia^9^ but only in recent years has CVA6 become one of the predominant serotypes in the country, as reported in the other studies mentioned previously. In fact, the emergence of CVA6 as the pathogen responsible for HFMD outbreaks has been observed long ago in other countries, dating back to 2008^21^, but not in Malaysia. This might be due to the limited number of studies on the characterization and identification of enterovirus types that caused HFMD in Malaysia, especially in Sabah.

Four sub-genotypes, B3, B4, C1 and C2 of the neurovirulent EV71 were found co-circulating and caused the 1997 HFMD outbreak in Peninsular Malaysia, while sub-genotypes B3 and C1 co-circulated in Sarawak in the same year^9^. Sub-genotype B3 was the predominant type that was associated with fatal cases in 1997^7^. Later in 2000, sub-genotypes B4, B5 and C1 co-circulated during the outbreaks in Peninsular Malaysia and Sarawak, where B4 emerged as the predominant sub-genotype^7,9^. From 2003 onwards, EV71 in Malaysia was mainly of sub-genotype B5, with minor contribution from sub-genotype C1 until 2005^7–9^. The predominance of sub-genotype B5 in Malaysia remains even after a decade, as shown in Ling et al. (2014)^10^. The EV71 strain in the present study was also classified as sub-genotype B5 but with the limited number of samples in this study, further investigations with a larger sample size are necessary to determine the prevalence of sub-genotype B5 in Malaysia.

Genotype B of CVA16 was identified in samples from Sarawak and Peninsular Malaysia from 1997 to 2014. Sub-genotype B2a was found circulating from 1997 to 2007 in Selangor, Kuala Lumpur and Johor, while sub-genotype B2c was found in Selangor from 2006 to 2007^11^. In 2014, CVA16 strains identified in samples from Seri Kembangan, Selangor were sub-genotypes B2b and B2c^10^. In Sarawak, several isolates from 1998 to 2000 were classified as sub-genotype B1^11^. Meanwhile, sub-genotype B2a was the main type of CVA16 circulating in the region in 1997–2003, 2005 and 2007 and sub-genotype B2c only emerged after 2005^11^. According to the sequence analysis of VP1 gene in this study, all CVA16 strains were classified as sub-genotype B1a, which has never been reported from Malaysia and is distinct from those isolated from Sarawak and Peninsular Malaysia. Phylogenetically, our CVA16 strains were close to the strains from Thailand, suggesting that they share a common ancestor. The first characterization of CVA16 and the identification of sub-genotype B1a in Sabah can shed some light on the genomics of HFMD-causing enterovirus in the region.

As in our study, EV71 and CVA16 have been identified circulating in several countries, including China, Japan, and Thailand^6^. Further, co-circulation of both CVA16 and EV71 serotypes during outbreaks has been reported in various countries, including Malaysia, Taiwan, and China^22^, which is a significant public health concern. It has been suggested that co-circulation of multiple serotypes of enterovirus may cause genetic recombination and co-infection, leading to increased risk of severe disease^23^.

In support of this notion, the present study found that HFMD mainly affected children under-five years of age, especially one-year-old children. This may be attributed to a decrease in maternal antibodies when they start weaning and more interaction with other children. A similar trend has been reported in other countries showing that HFMD cases were mostly found in children under the school-age^24^. However, infants less than one-year-old had a lower infection rate, presumably due to the lack of contact with other children or the protective effect of maternal antibodies from breast milk^25^. In agreement with other studies^24,26^, this study also revealed male predominance in HFMD cases, possibly attributed to the different degree of attention and treatment given by caregivers between boys and girls, especially in Asian countries. Caregivers are more likely to seek medical care for boys than girls^24^. On the contrary, in Singapore and Taiwan, gender did not significantly affect the infection rate^27,28^.

As shown in this study, other countries in Asia, such as China^29^, Singapore, and Japan^7^, also observed an increment of more than two-fold in the number of cases throughout the last decade. Besides, this study also found that areas with high population density and more developed economies, such as Kota Kinabalu and Penampang had high number of HFMD cases, which is consistent with previous studies^30^. This may be attributed to the economic development and urbanization in these areas leading to more frequent communication and close contact with individuals, resulting in a higher risk of HFMD transmission^31^. However, high incidence rates were also found in low populated rural areas, such as Sipitang and Kuala Penyu, which may be due to inadequate sanitary conditions, lack of clean water supply, poor hygiene practices and limited knowledge on the disease^32^.

In conclusion, HFMD in Sabah is often overlooked, and the disease warrants attention since the number of cases has increased remarkably over the last five years. Knowledge of the epidemiological and molecular characteristics of HFMD in Sabah is essential to assist in developing appropriate prevention and effective treatment strategies. This will contribute to the overall success of reducing the burden of communicable diseases in Malaysia. This study, for the first time, described the epidemiological features of HFMD in Sabah and investigated the enterovirus serotypes that might be responsible for the disease in the area. Due to the limited number of samples in this study, further studies are necessary to include more HFMD samples from different areas of Sabah for molecular analysis to gain a better understanding of the etiologic causes of HFMD in the region.

## Materials and methods

### Ethics statement

HFMD notifications are captured under the Public Health Infectious Disease Surveillance System for the purpose of analysis of data and determining the epidemiological pattern and characteristics. The study was registered under the National Medical Research Registry and the study protocol was approved by the Medical Ethics and Research Committee, Ministry of Health Malaysia (NMRR-20-3286-56537). Permission was obtained from Director-General of Health Malaysia to publish this article (Ref. no. 800-4/4/1 Jld.91(56) dated February 4, 2021). All procedures were performed in accordance with the relevant guidelines, protocols, and regulations of the Ministry of Health, Malaysia (https://www.moh.gov.my/moh/resources/auto%20download%20images/589d71f714d23.pdf). Clinical samples were taken as part of outbreak control measures and informed consents were obtained from guardians of the children. All information was kept anonymous.

### Study site

**Figure 5:**
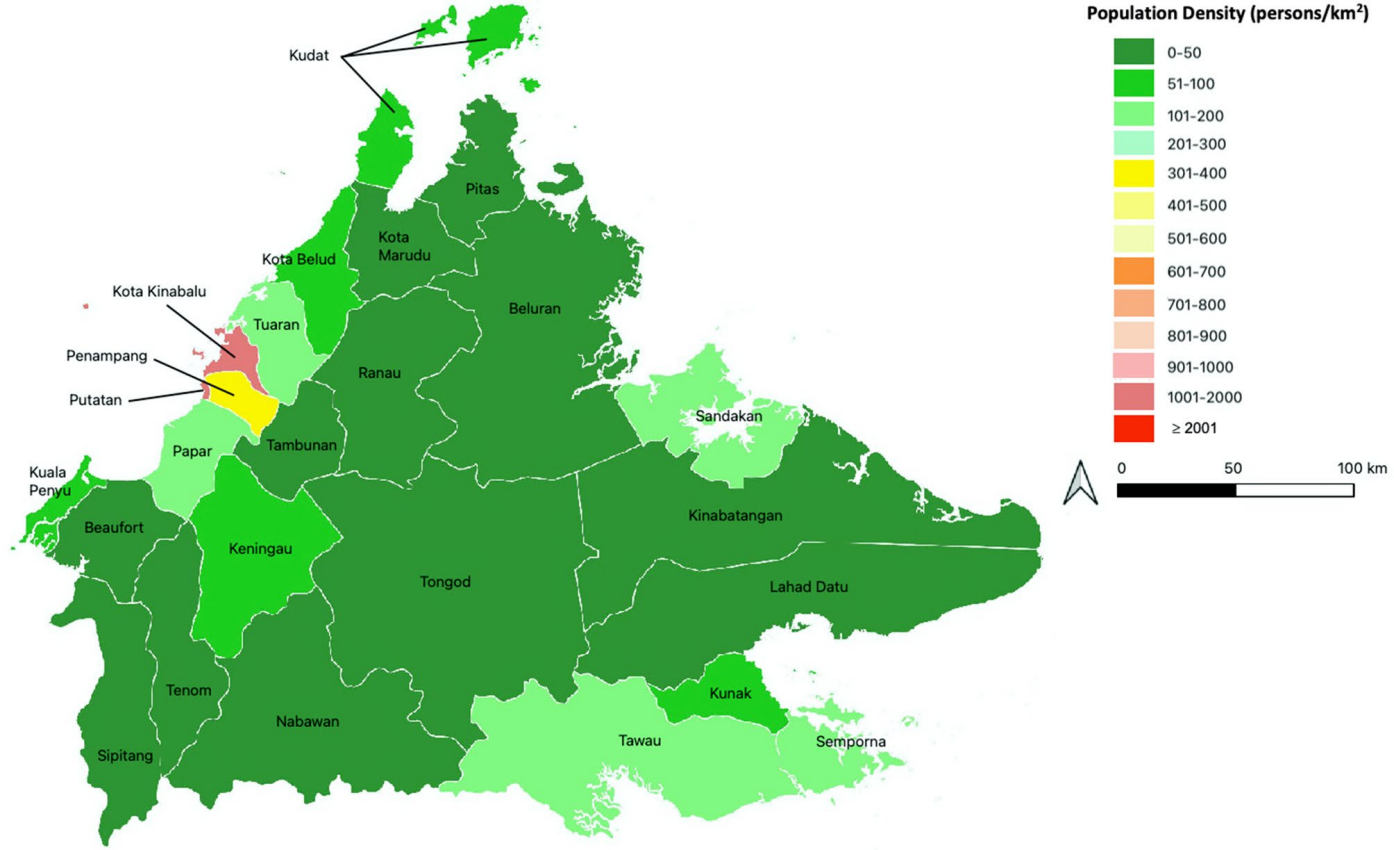
Map of Sabah, showing its 25 districts, which are colored according to their population density, expressed as number of persons per square kilometer. The map was generated using QGIS version 3.18.1 Zurich (https://www.qgis.org/).

Sabah is a region in Malaysia, located at the northern portion of the Borneo island. The region, with a geographical area of 73,904 km^2^, consists of 25 districts^33^, with an estimated population of 3.9 million in 2019^34^ (Fig. 5). Kota Kinabalu is the capital city of Sabah, and it has the highest population density of 1397 per square kilometer. Sabah has a relatively low urban population compared to other Malaysian states, where only 54% of Sabah’s population live in urban areas^33^. Sabah has an equatorial climate with high humidity and high annual rainfall.

### Epidemiological data

From January 2015 to December 2019, a total of 17,574 clinically diagnosed and PCR-confirmed HFMD cases in Sabah were reported and recorded in a Malaysian public health disease surveillance system called *eNotifikasi* (also known as Communicable Disease Control Information System; http://enotifikasi.moh.gov.my). Data of all reports of HFMD cases were extracted from the database, including demographic information, date of onset, date of diagnosis, and classification of transmission.

### Sample collection for molecular analysis

Thirteen children ranging from one to eight years old with clinical symptoms of HFMD, such as fever, mouth ulcers, maculopapular rashes, and vesicles on palms and soles, were included in the study. Twenty-one samples from these children, including rectal, mouth, vesicle, and sole swabs, were collected from Beaufort District Health Office, Beaufort, Sabah, from August 9, 2018, to August 13, 2018. The swabs were kept in viral transport medium (VTM), labeled, and refrigerated before being transported to the Borneo Medical and Health Research Centre in Universiti Malaysia Sabah for molecular analysis.

### Sample processing

The collected samples were vortexed and filtered through a 0.22 µm syringe filter. The filtrate of each sample was stored in a 2 mL screw-cap tube, labeled, and kept at − 80 °C until further analysis.

### Viral RNA extraction and first-strand cDNA synthesis

Viral RNA was extracted from samples using the QIAamp Viral RNA Mini Kit (Qiagen, Germany) according to the manufacturer’s instructions. The RT-PCR procedure for each amplification was performed in a 10 µL reaction mixture with 2.5 µL of the extracted RNA template, cDNA primers AN32, AN33, AN34, and AN35 (Table 3), and the SuperScript III First-Strand Synthesis System (Invitrogen, USA). The mixture was incubated at 50 °C for 50 min, then at 85 °C for 5 min before storing the cDNA synthesis reaction at − 20 °C for further analysis.

**Table 3:** Primers used for cDNA synthesis, PCR amplification, and sequencing.

Primer	Sequence (5′–3′)	Gene	Location
AN32	GTYTGCCA	VP1	3009–3002
AN33	GAYTGCCA	VP1	3009–3002
AN34	CCRTCRTA	VP1	3111–3104
AN35	RCTYTGCCA	VP1	3009–3002
MD91	CCTCCGGCCCCTGAATGCGGCTAAT	VP4	444–468
OL68-1	GGTAAYTTCCACCACCANCC	VP4	1178–1197
EVP4	CTACTTTGGGTGTCCGTGTT	VP4	541–560
224	GCIATGYTIGGIACICAYRT	VP3	1977–1996
222	CICCIGGIGGIAYRWACAT	VP1	2969–2951
AN89	CCAGCACTGACAGCAGYNGARAYNGG	VP1	2602–2627
AN88	TACTGGACCACCTGGNGGNAYRWACAT	VP1	2977–2951

### Enterovirus detection using semi-nested reverse-transcription polymerase chain reaction (snRT-PCR)

Enteroviral RNA was detected in samples by snRT-PCR of the VP4–VP2 region. The first primer pair, MD91-OL68-1 (Table 3), was used to amplify the partial 5’ non-coding region, VP2, and complete VP4. 3 µL of RNA was used in the PCR using the AccessQuick RT-PCR System (Promega, USA). The PCR condition consisted of 1 cycle of initial denaturation at 95 °C for 2 min, 40 cycles of denaturation at 95 °C for 30 s, annealing at 55 °C for 30 s, and extension at 72 °C for 1 min, followed by 1 cycle of final extension at 72 °C for 7 min.

snRT-PCR with the second primer pair, EVP4-OL68-1 (Table 3), was performed by using 2 µL of the PCR product under the following PCR condition: 1 cycle of initial denaturation at 95 °C for 6 min, 40 cycles of denaturation at 95 °C for 30 s, annealing at 55 °C for 30 s, and extension at 72 °C for 1 min, followed by 1 cycle of final extension at 72 °C for 7 min.

### Enterovirus serotyping using consensus degenerate hybrid oligonucleotide primer (CODEHOP)

The enterovirus VP1 gene sequence was amplified using a CODEHOP protocol described by Nix, Oberste, and Pallansch (2006)^35^. Briefly, the cDNA generated by the four different primers (AN32–AN35) was used for the first round of PCR with primers 224 and 222 (Table 3). The condition for the first PCR consisted of 1 cycle of initial denaturation at 95 °C for 6 min, 40 cycles of denaturation at 95 °C for 30 s, annealing at 42 °C for 30 s, and extension at 60 °C for 1 min. The first PCR generated a product of 992 bp, which was used for the second round of PCR with primers AN89 and AN88 (Table 3) for nested amplification. The condition for the second PCR consisted of 1 cycle of initial denaturation at 95 °C for 6 min, 40 cycles of denaturation at 95 °C for 30 s, annealing at 60 °C for 30 s, and extension at 72 °C for 30 s, followed by 1 cycle of final extension at 72 °C for 5 min. The second PCR generated a product of 375 bp.

### Sequence analysis

Partial nucleotide sequences of the VP1 gene of CVA16 (254 bp) and EV71 (359 bp) were used for phylogenetic analyses. The amplified DNA of each positive sample was sequenced using the BigDye Terminator v3.1 Cycle Sequencing Kit (Applied Biosystems, Foster City, CA) according to the manufacturer’s instructions, and the product was run on an ABI Prism 3100 Genetic Analyzer (Applied Biosystems). Nucleotide sequences of VP1 were checked against the NCBI database by BLAST to find the enterovirus serotype with the highest identity. The nucleotide sequences of other CVA16 and EV71 strains were extracted from GenBank. Multiple sequence alignment was performed using Clustal W, and phylogenetic trees were constructed using MEGA 6.0, applying the neighbor-joining method based on the Tamura–Nei substitution model^36^. Bootstrap analysis of 1000 replicates was conducted to determine the significance of the branching of the constructed tree. Nucleotide sequences analyzed in this study have been submitted to the DNA Data Bank of Japan (DDBJ).

### Data analysis

Data analysis was performed using Microsoft Excel for Mac 2017 (Version 15.30). QGIS v3.14.1 (Pi) was used to generate spatial maps of HFMD cases in Sabah.
